# Intervention Fidelity of Telephone Motivational Interviewing On Physical Activity, Fruit Intake, and Vegetable Consumption in Dutch Outpatients With and Without Hypertension

**DOI:** 10.1007/s12529-022-10076-8

**Published:** 2022-03-28

**Authors:** Ilse Mesters, Hilde M. van Keulen, Hein de Vries, Johannes Brug

**Affiliations:** 1 Department of Epidemiology, Maastricht University, PO Box 616, 6200 MD Maastricht, The Netherlands; 2 Department of Health Promotion, Maastricht University, PO Box 616, 6200 MD Maastricht, The Netherlands; 3 Department of Child Health, TNO, PO Box 3005, 2301 DA Leiden, The Netherlands; 4 National Institute for Public Health and the Environment, PO Box 1, 3720 BA Bilthoven, The Netherlands; 5 The Amsterdam School of Communication Research, University of Amsterdam, PO Box 7057, 1007 MB Amsterdam, The Netherlands

**Keywords:** Fruit consumption, Vegetable consumption, “Activities, physical”, Motivational Interviewing, Fidelity, Behavior change, Fidelity-effectiveness association

## Abstract

**Background:**

In theory, Motivational Interviewing (MI) fidelity should be associated with client outcomes. Nevertheless, this fidelity-effectiveness association is rarely investigated. This study evaluated the extent to which Telephone Motivational Interviewing (TMI) fidelity is associated with change in self-reported physical activity (PA), fruit intake, and vegetable consumption.

**Method:**

Adults in primary care (45–70 years) participated in a study that compared the effect of tailor print communication, telephone motivational interviewing (TMI), and a combination of the two on PA, fruit intake, and vegetable consumption. MI fidelity was assessed using the behavioral coding method “Motivational Interviewing Treatment Integrity Code (MITI)” in 409 randomly selected audio-recorded sessions, representing 232 participants of the TMI group. The associations between MI fidelity scores and the behavioral changes from baseline to 47-week follow-up were examined using backward multiple linear regression analyses (adjusted for covariates).

**Results:**

A significant and positive association between the percentage of MI adherent responses and improvements in PA and fruit consumption was found with respectively a small and medium effect size. The global rating “Spirit” (which resembles an all-at-once appraisal of the interviewer’s MI competence) was significantly, but inversely associated with progress in vegetable intake with a medium effect size.

**Conclusion:**

The finding that relatively lower MI competency was associated with higher vegetable consumption went against our expectations. Findings suggest that practicing MI-consistent skills was beneficial in promoting PA and fruit consumption, but moderated vegetable intake. This study contributes to the scientific confidence that TMI enables change in PA and fruit intake.

## Introduction

The number of older people is increasing at a greater rate than any other age group [1]. Adults in their middle to late years are expected to live longer, but they are not necessarily living healthier lives. Unhealthy lifestyles put people at risk for a variety of cardiovascular diseases (CVD), including coronary heart disease, cerebrovascular disease, and peripheral vascular disease [2]. Meeting the national guidelines for a healthy diet [3] and PA [4] may reduce the risk of cardiovascular morbidity and mortality [5] [6]. Therefore, it is recommended that adults with an unhealthy lifestyle be referred to behavioral change programs that encourage PA and a healthy diet in order to prevent CVD [7].

According to the Dutch recommendations for PA, adults should engage in moderate-intense physical activity for at least 30 min on most days (at least 5) of the week [8]. In the Netherlands, fruits and vegetables have separate dietary recommendations due to differences in consumption circumstance and meals, as well as their associations with health and disease [9]. The nutritional advice for daily fruit and vegetable intake is two pieces of fruit (at least 200 g) and at least 150–200 g of vegetables [8].

As a large portion (about 60%) of the Dutch adult population is inactive [10, 11] and more than 85% does not meet the aforementioned diet norms [11, 12], there is a need to find efficient ways of delivering interventions that motivate people to change these behaviors. Telephone Motivational Interviewing (TMI) is a motivational approach that can reach a large number of people [13]. MI is a type of counseling that aims at stimulating health-related motivation and behavior change. MI assumes that clients know what is best for them, and counselors are there to assist them in making positive changes in their lives. It incorporates two components, which are categorized as principles and particular counseling behaviors. These components and their sub-elements constitute the basic criteria for the assessment of MI fidelity. Principles concern the overall spirit of MI, which are divided into three categories [14]: collaboration, evocation, and autonomy support. A collaborative counselor avoids adopting an authoritarian attitude and, instead, appreciates the client’s expertise and perspectives in a partner-like relationship. Instead of trying to advise or teach the client, an evocative counselor strives to elicit the client’s motivation for change, for example, by inviting the client to verbalize the reasons for change (known as change talk). A counselor who supports the client’s autonomy acknowledges that the responsibility for change lies with the client, and thus tries to avoid imposing the motivation for change. Furthermore, four guiding principles underlie the overall spirit of MI. The first is expressing empathy: an empathic counselor tries to understand and appreciate the client’s perspective. A fundamental aspect involved in this is skillful reflective listening. The second principle is developing discrepancy: clients are encouraged to change by letting them see the discrepancy between their present unhealthy behavior and important personal goals or values. The third is rolling with resistance: the counselor must avoid advocating for change because this frequently leads to the client defending the opposing viewpoint. Thus, rather than opposing resistance, the counselor acknowledges it as being natural and understandable. Supporting self-efficacy is the fourth guiding principle; this is the client’s belief in the possibility of change, and it functions as a motivator for change [15].

Five counseling behaviors appear to be useful throughout the MI process and should be used to support MI’s spirit and underlying principles. Four of them (i.e., asking open questions, listening reflectively, affirming, and summarizing), which are derived from client-centered counseling, are used by the MI counselor in the service of the fifth MI-specific counseling behavior (i.e., eliciting change talk) [15]. Examples of clients’ change talk are recognizing the drawbacks of the current situation, expressing optimism about change, or expressing the intention to change. The principles and counseling behaviors of MI are often applied in two phases, first to build intrinsic motivation for change, and second—when the client is willing and able to change—to enhance the commitment to change and developing a change plan [15].

MI principles and counseling behaviors are practice based and several of them overlap with existing behavioral change theories (entailing variables as attitude, self-efficacy, social influence, intention to change). These theoretical concepts were used to design the content of our TMI counseling protocol (Example Appendix 1) [16]. For instance, discussing the pros and cons of whether or not to change (decisional balance) is practiced by MI counselors and is part of our counseling script [16–18]. The content is about what is being said and communication skills concern how a topic is addressed. Therefore, for our study’s reliability and internal validity, it was important not only to assess whether, for instance, weighing pros and cons lead to changes in lifestyle behavior, but also to evaluate whether the fidelity with which MI was executed may have implications for our study’s outcomes. The Motivational Interviewing Treatment Integrity tool (MITI 3.0) was used to examine the qualifications of our MI counselors, who were judged to have beginner proficiency [19]. This suggests that the next step, to link MI fidelity to change in PA, fruit intake, and vegetable consumption in older adults, is appropriate.

So far, there have been few studies that have evaluated the fidelity of MI delivery and have linked MI components to outcomes [20, 21]. Moreover, the evidence that is available is mixed, with positive, negative, and no evidence of an association [22–24].

To better understand our treatment effects, this study examines not only whether the defined (behavioral) concepts that guided the content of the TMI intervention and several background variables are associated with lifestyle behavior changes [25–27], but also whether the fidelity with which counselors apply MI is associated with behavioral change.

## Methods

### Description of the Original Study Design

Our trial originally compared the effects of four computer-generated tailored print communications (TPC), four telephone-delivered MI sessions (TMI), and a combined intervention (two TPC letters and two TMI sessions), with no intervention on PA, fruit intake, and vegetable consumption [25]. TPC and TMI are both individualized interventions, in the sense that they provide feedback based on characteristics of the individual. Though both types of intervention focus on the individual, there are differences between them. TPC lacks the relational support and interactional aspects of TMI. This interpersonal nature may make TMI more individualized than TPC. However, because the latter is not person-delivered, it is easier to reach larger groups of people with TPC than with TMI [28]. On the other hand, TMI can be more expensive and time consuming than TPC because it requires the use of skilled counselors [27].

In the TPC intervention, participants complete a survey with questions on their health, behavior, and presumed behavioral determinants, and the results are saved in a data file. The data are linked to a message library that contains all of the health education messages that may be required, i.e., messages that are appropriate for all of the various survey responses. Computer software with if-then algorithms is used to connect the individual survey responses to the messages. Next, the computer program generates feedback for each participant, which is then printed and mailed to the participants [25]. The surveys, messages, and algorithms are based on expert input, behavioral theories, and empirical evidence.

TMI entails a counseling approach, as described, conducted during a scheduled telephone appointment, that can help people find the motivation to make positive health decisions and commitment to change in order to achieve a specific health goal [13]. TMI addresses similar behavior change component as TPC, i.e., decisional balance, social influence, self-efficacy, intention to change, and action planning.

The participants in the TMI group will make up the study population in the remainder of this work in which it is examined whether fidelity of the TMI intervention is associated with change in PA, fruit intake, and vegetable intake.

### Participants and Attrition

It was anticipated that 50% of those invited would participate, of whom 50% would fail to meet at least two of the three guidelines. Dropout was estimated at 20%. Based on power calculations, a total sample size of 1,600 participants (400 per study group) was needed at baseline using a small effect size of *d* = 0.3, a power of 90%, a two-tailed alpha of 1% to correct for multiple testing, an intraclass correlation of 0.02, and an average sample size of 70 participants per general practice (GP) [25].

**Fig. 1:**
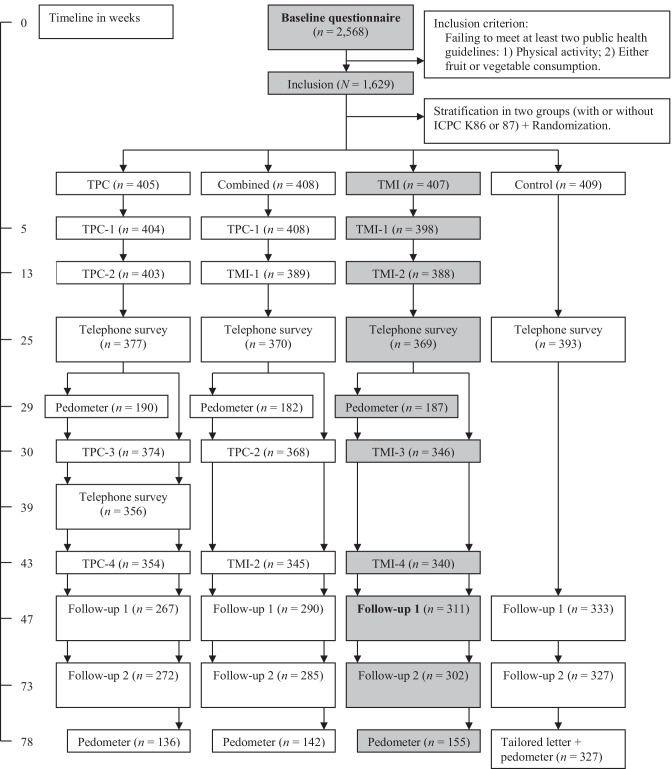
Study design and timeline. Notes. ICPC International Classification of Primary Care; K86 or K87 hypertension without or with organ damage, respectively; TPC tailored print communication; TMI telephone motivational interviewing; Combined, combination of TPC and TMI

Adults (*n* = 6,420 outpatients) from 23 Dutch general practices (GPs) were randomly selected from the Research Network Family Medicine Maastricht database using a computer program [25]. About half of the adults chosen should be men and only one person per address should be selected. Inclusion criteria were as follows: (1) aged 45–70 years; (2) about 50% diagnosed by their GP as hypertensive according to the International Classification of Primary Care (ICPC code K86 or K87 for hypertension without or with organ damage respectively) [29]; (3) not participating in other studies according to the GP database; (4) failing to meet at least two of the three guidelines. Hypertension status was included to see if having a risk factor for CVD (disease awareness) moderated the intervention effects. Following the GP’s suitability check (e.g., not able to speak or read Dutch, physical and mental disabilities) [11], 5,545 people received an invitation letter outlining the study’s setup, and 2,881 of them returned a signed written informed consent form. Of the 2,568 responders to the lifestyle assessment, 1,629 were included in an RCT if they failed to meet at least two of three Dutch public health guidelines (for PA and either fruit or vegetable intake) (see Fig. 1). A more detailed description of the baseline (non-) responders was published elsewhere. The obtained population was a good reflection of the selected population [11]. After the baseline assessment, all parties were aware of the treatment groups. Researchers had no in-person contact with participants (measurements were filled in at home). Measurement dropout over time was found to be unrelated to age, sex, hypertension, and region, and was significantly higher in the TPC group (alone or combined) than in other participant groups, particularly from week 47 onwards [26]. Possible bias effect of dropout was adjusted for in the effect analyses by including all dropouts and all predictors of dropout into the analyses of each behavioral outcome [30].

### TMI Intervention Logistic

The TMI group participants (*n *= 407) received four telephone counseling sessions, two of which focused on PA and the other two on both fruit and vegetable intake in tandem. The participants in the TMI study group were followed from the baseline to the first posttest at 47-week follow-up. In the first and third sessions, participants could choose the order of the conversation topics of the two subsequent TMI counseling session (“agenda setting”). For example, in case they preferred to discuss PA in the first session, then they talked about fruit and vegetable consumption in the second session, and vice versa. This procedure was followed in sessions 3 and 4, as well. After participants had returned the baseline questionnaire by mail, the counseling sessions were held at 5, 13, 30, and 43 weeks. In total, 1,472 counseling sessions were conducted in the TMI group. A (computer-generated) random sample of 409 sessions was selected from each time point (about a hundred per week: weeks 5, 13, 30, and 43, respectively) representing 232 participants who are involved in the current study. Table 1 includes the participant characteristics.

**Table 1:** Means (SD) or percentages of baseline variables (used as covariate) for the subsample and total RCT sample

**Variables (minimum–maximum value total sample)**		**Selected sample (n = 232)**	**Total sample (n = 1,629)**
Sex–% male		56.9	55.5
Age (45–70 years)		57.7 (7.3)	57.1 (7.1)
Native country—% the Netherlands		94.4	94.8
Region—% southern Limburg		65.1	62.4
Season at baseline	*% spring; summer*	76.3; 11.6^†^	74.9; 9.5
	*% autumn; winter*	0.9; 11.2^†^	2.4; 13.2
Education level—% low; intermediate; high*		48.7; 24.8; 26.5	54.0; 23.3; 22.7
Marital status—*% married or living together*		84.8	79.8
Work situation—*% paid job*		46.3	48.5
Hypertension—*% hypertensive*		52.6	51.8
Diabetes—*% diabetic*		12.6	10
Perceived stress	*% less than normal; normal*	19.0; 48.5	14.9; 50.2
	*% a little more; too much*	18.2; 14.3	22.6; 12.3
CVD family history—*% no family history*		39.6	41.5
Body mass index (kg / m^2^; 15.2–46.7)		27.7 (4.5)	27.4 (4.6)
Smoking behavior—*% nonsmokers*		81.3	78.3
Alcohol consumption—% non-drinkers; drinkers meets guideline; does not meet guideline**		38.1; 48.9; 13.0	38.1; 48.1; 13.8
Saturate fat intake score (2.0–37.0)***		17.6 (5.8)	17.8 (5.9)
PA	Hours/week moderately intense (0–20.2)	4.6 (4.0)	4.7 (3.8)
	Awareness—*% overestimating PA*	59.3	59.6
	Attitudes—pros (13–65); cons (11–55)****	49.6 (7.1); 39.2 (6.4)	49.1 (7.3); 38.6 (6.6)
	Social support (5–25); modeling (3–15)****	14.8 (4.3); 9.7 (2.8)	14.5 (4.0); 9.5 (2.6)
	Self-efficacy expectations (11–55)****	36.8 (7.6)	36.8 (7.6)
	Number of action plans (0–6)	2.2 (1.0)	2.2 (1.1)
	Habit (3–15)****	10.3 (2.9)	10.6 (2.7)
	Stages of change (1–6)	4.1 (1.8)	4.1 (1.9)
Vegetable intake	Grams of vegetables/day (0–494.9)	165.2 (82.1)	165.2 (82.4)
	Awareness—*% overestimating intake*	85.3	85.6
	Attitudes—pros (8–40); cons (8–40)****	30.0 (4.5); 31.0 (4.7)	29.7 (4.3); 30.8 (4.7)
	Social support (5–25); modeling (3–15)****	14.2 (4.1); 10.2 (2.0)	14.0 (4.0); 9.9 (1.9)
	Self-efficacy expectations (9–45)****	34.0 (5.6)	34.2 (5.6)
	Number of action plans (0–6)	2.2 (1.0)	2.2 (1.0)
	Habit (3–15)****	12.1 (2.6)	12.1 (2.4)
	Stages of change (1–6)	4.7 (1.8)	4.7 (1.8)
Fruit intake	Servings of fruit/day (0–8.9)	2.1 (1.6)	2.1 (1.6)
	Awareness—*% overestimating intake*	55.2	56.9
	Attitudes—*pros (8–40); cons (4–20*)****	29.0 (4.8); 15.2 (2.4)	28.5 (4.6); 15.0 (2.6)
	Social support (5–25); modeling (3–15)****	13.4 (4.0); 9.4 (2.1)	13.3 (4.0); 9.4 (2.0)
	Self-efficacy expectations (10–30)****	37.7 (6.1)	37.6 (6.6)
	Number of action plans (0–5)	1.8 (0.8)	1.9 (0.9)
	Habit (3–15)****	10.2 (3.2)	10.1 (3.3)
	Stages of change (1–6)	4.0 (1.9)	4.1 (2.0)

*PA* physical activity

*Education level is low in participants with less than secondary or vocational education; intermediate in participants with secondary through pre-university education; and high in participants with professional or university education (European Commission 2007; Ministerie van Onderwijs 2003; UNESCO 1997); **Participants were classified as meeting the alcohol consumption guideline if they consumed fewer than 3 glasses a day (men) or fewer than 2 glasses a day (women) [8]; ***One fat point represents about 2 g of daily saturated fat intake; ****Item range = 1–5, the values are sum of scores; a higher score is positively related to the health behavior. ^†^Because percentages are rounded off, sum total does not equal 100%

Counselors were 16 bachelor’s and master’s level students in Health Sciences and Psychology [19]. They received six 3-h training sessions by two certified trainers after which they had to perform one TMI conversation with adequate integrity according to the 1-Pass Coding System for motivational interviewing [31]. A protocol was developed for each interview and provided to counselors to support them in applying MI, with the aim of ensuring treatment fidelity. The protocol steps were as follows: introduction, assess current behaviors and progress, discuss the public health guideline, assess and enhance motivation and self-efficacy for behavior change, assess readiness to change, summarize and close the session. Due to practical constraints, different therapists delivered the four sessions to one participant. Counselors read the standardized communication logs from prior sessions with a specific participant to prepare themselves for their session.

### Measurement

#### MI Fidelity

Counselors’ MI fidelity was assessed with a behavioral coding system called the MITI 3.0 (available at http://casaa.unm.edu/download/miti3.pdf) [32]. The MITI 3.0 is designed to assess two components: the global scores are meant to capture the coder’s global impression of the entire communication interaction and the behavior counts, which require the coder to count particular practitioners’ counseling behaviors (such as “the use of open questions”).

The global score requires the coder to assign a single number from a 5-point scale (1 = low to 5 = high) to capture the rater’s overall judgment of how well or poorly the counselor meets the intent of the scale. Behavioral anchors for each point on a scale guide the coding. The *global counselor scores* are calculated by the average of (1) *empathy* (counselor attempts to find out what the client feels or thinks), (2) *direction* (counselor maintains an appropriate focus on a specific target behavior), and (3) *spirit* (an all-at-once judgment of the interviewer MI competency). The latter is the average of *evocation* (counselor understands that change resides within the client), *collaboration* (counselor works towards mutual understanding), and *autonomy* support (counselor actively fosters client’s perception of choice).

The purpose of behavior counts is to capture specific counseling behaviors regardless of how they fit into the overall perception of the interviewer’s use of MI. The expressions of the counselors are counted regarding giving information, questions (open vs. closed), reflections (simple vs. complex), MI adherent behaviors (e.g., asking permission, emphasizing control, affirming or supporting), and MI nonadherent expressions (e.g., advising without permission, confronting or directing). Summary scores are calculated to obtain the percentage of open questions, the percentage of complex reflections, the reflections/questions ratio, and the percentage of MI adherent expressions.

Coders rated the sessions independent of knowing about the outcome data. Seven master’s level students/graduates in Health Promotion received 40 h of coding training [19]. Coders were trained by a Dutch certified instructor. The training included readings, videotapes, and brief discussions. Coders used a computer to listen to the recorded interviews. They assessed the counselors’ competence with a printed version of the MITI 3.0.

In total, 94 sessions (28%) were double coded. Inter-rater reliabilities were fair to good, except for the global score on direction (ICC .35), the % complex reflection (ICC .23), and the number of simple reflection (ICC .32) [19]. The MI fidelity for global clinician scores (direction, empathy, spirit) and the percentage of complex reflections exceeded the MITI (3.0) threshold for beginning proficiency (MI cutoffs: mean > 3.5 on a 5-point scale and > 40%). Lower scores were found for the percentage of open questions (< 50%), the reflections-to-questions ratio (1), and the percentage of MI adherent responses (> 90%) [19, 32]. Beginning proficiency level with core client-centered counseling was considered satisfactory [19].

#### Lifestyle Behaviors

To assess change in PA and fruit and vegetable consumption, postal surveys were conducted, supplemented with two postal reminders at the baseline and 47-week follow-up.

The 28-item modified CHAMPS Physical Activity questionnaire assessed the frequency of a PA activity (times per week) and its duration (hours per week) and intensity (e.g., walking in a leisurely vs. brisk manner) concerning a typical week during the past four weeks [33]. Activities included, for example, walking leisurely or briskly and doing light or heavy housekeeping. Based on the PA compendium by Ainsworth et al. [34], metabolic equivalents (METs) and the oxygen cost of energy expenditure were determined for each activity. The recommended cutoff to define at least moderate-intensity activities is ≥ 3 MET, which was used to calculate the total number of weekly PA hours with at least a moderate intensity. The modified CHAMPS has been validated with cardiorespiratory fitness (VO_2_ maximum) estimated by a submaximal treadmill test [33]. Others reported adequate test-retest reliability for activities ≥ 3 METs and revealed an adequate association with accelerometer minutes of corresponding intensity [35].

A 16-item short fruit and vegetable questionnaire assessed frequency (days per week) and quantity of daily vegetables (cooked and raw), and daily fruit consumption (fruit juice, tangerines, other citrus fruits, apples or pears, bananas, and other fruits) concerning a typical week during the past four weeks [36]. The outcome was the quantity of daily consumption; fruit consumption in servings/day (one piece of fruit is considered one serving); and intake of vegetables in grams/day.

Additionally, a range of possible covariates were assessed including patient characteristic and potential cognitive-behavioral determinants of the target behaviors (i.e., awareness, pros and cons, social support, modeling, self-efficacy expectations, action plans, habit strength, and stages of change) (see Table 1). These cognitive-behavioral variables represent the I-Change Model [37].

Patient characteristics included at baseline were, i.e., sex, age, highest completed level of education, marital status, work situation, ethnicity, region in which participants were living, and season in which the baseline questionnaire was completed.

Other variables were PA, fruit intake and vegetable consumption, smoking behavior, alcohol consumption, saturated fat intake, and health variables (i.e., hypertension, presence of diabetes, family history of CVD, stress, and body weight and height to calculate the body mass index (BMI)). A description of the questionnaire was published [30].

### Data Analysis

Of the selected sample of 409 20-min session fragments, 336 were coded (representing 232 TMI participants). Computer recording failure or bad recording quality hampered coding. If more than one interview of a participant was coded, then the indicator of MI fidelity per participant was the average of the MITI 3.0 values across the interviews.

Handling missing values and checking data for outliers were discussed elsewhere [30]. The primary outcomes were checked for normality. Because of positive skewness, fruit in servings a day and PA in hours a week were square root transformed at baseline and at 47-week follow-up for all analyses. The heteroscedasticity of residuals was checked and not found. Data were examined for multi-collinearity by inspecting the variance inflation factor (VIF) of each predictor in a multiple regression model with all baseline variables as predictors. No VIFs above 10 were found, indicating the absence of multi-collinearity [38]. Impact of measurement dropout over time was analyzed with mixed regression analysis (using PQL estimation, ML-wiN software).

SPSS version 15.0 was used to describe descriptives and frequencies. To check for baseline differences (*α* = .05), a backward multiple logistic regression analysis was conducted comparing the randomly selected sample of participants from the TMI group (*n* = 232), whose counseling sessions were selected to the other participants that were not included. The dependent variable was “selection” and independent variables were sociodemographic variables, lifestyle and health variables, and PA, fruit intake, and vegetable consumption at baseline.

The relation between MI fidelity and behavior change was examined with backward multiple linear regression analyses. No multilevel analysis was done, because there was no evidence for general practice effects and because participants were not nested within interviewers or coders. For each outcome behavior (PA in hours/week, intake of fruit in servings/day, and consumption of vegetables in grams/day), the dependent variable was the difference of the outcome value between baseline measurement and the follow-up at week 47 [39]. Patient characteristics, potential cognitive-behavioral determinants, other lifestyle and health variables, baseline behavior of other outcomes, and choice of the order of conversation topic were used as covariates. The inclusion of these covariates improves the power and precision of effect testing and estimation due to reduced residual outcome variance [39]. In the original study, the efficacy was examined of participants receiving a pedometer by sending a random half of the participants in the three intervention groups a pedometer before the third intervention component (week 29), the rest received this instrument after the 47-week follow-up. Whether participants received a pedometer during the intervention was included as an additional covariate in the analyses of the relation between MI fidelity and behavior change. MI fidelity summary measures (the three global clinician ratings, the percentage of open questions, the percentage of complex reflections, the percentage of MI adherent expressions, and reflection to question ratio) were included as independent variables. Non-significant independent variables and covariates were excluded from the model; manually one by one, the variable with the highest *p*-value was excluded first. The aim was to only to show the relevant predictors. However, hypertension and having received a pedometer during the intervention period remained in all models because these were used in the randomization procedures. Educational level, age, and sex were also kept in all models because these were used as selection variables or in hypotheses in the original study. Because of the explorative nature of the analyses, an alpha of .05 was used to exclude variables from the model. In view of multiple testing (i.e., diverse measures of fidelity were used as predictors), results with *p* between .05 and .01 must be interpreted with caution.

Cohen’s *f*^2^ was used to calculate the effect size. This is the most common method for calculating the effect size in a multiple regression model where both the independent and dependent variables are continuous. Cohen’s *f*^2^ is categorized as small (≥ 0.02), medium (≥ 0.15), or large (≥ 0.35).

## Results

In the selected TMI sample, 232 participants finished the baseline data collection and 194 (84%) completed the first follow-up. Logistic regression showed that slightly more participants in the selected sample were living together at baseline (84.9%) than were participants in the total sample (79%; OR = 1.49, 95% CI = 1.02 to 2.18). However, this result may be due to multiple testing and was not significant at the 1% level, implying a low risk of selection bias.

A disadvantage of linear regression analysis is that participants who have dropped out at 47 weeks after the baseline were excluded from the analyses (see the coming section about the relationship between MI fidelity and behavior change). Hence, a secondary backward logistic regression analysis was conducted to examine whether MI fidelity summary scores were related to dropout at the 47-week follow-up, corrected for sociodemographic variables, cognitive-behavioral determinants, lifestyle and health variables, and baseline outcome behaviors. These results revealed that MI fidelity summary scores were not related to dropout at the 47-week follow-up, indicating a low risk of selection bias (all *p* > .20).

### Behavior Change

The selected TMI sample (n=232) significantly (*p*<.01) increased their level of moderately intense PA from 4.65 hours a week (sd =3.97) at baseline to 5.45 hours a week (sd =4.37) at follow-up 1. At baseline, participants consumed 2.12 (sd =1.64) servings of fruit a day, whereas they had an intake of 2.83 daily servings (sd =1.99, *p*< .05) at follow-up 1. The vegetable intake was 165 grams/day at baseline (sd =82) and 184 grams (sd=78) at the first follow-up, *p*<.05.

### Association between MI Fidelity and Behavior Change

The global ratings (e.g., spirit, direction, and empathy) and the behavioral counts (e.g., percentages of open questions, complex reflections, MI adherent responses, reflection to questions ratio) were used to evaluate the relation between MI fidelity and change in PA, fruit, and vegetable consumption (Table 2).

**Table 2:** Beta’s (*B*), standard errors (SE), 95% confidence intervals (CI), *p* values, standardized beta’s (Beta), and *f*^*2*^ of predictors (covariates) of difference between baseline and 47 weeks follow-up values on the outcome behavior*

**Outcome variables**	**Predictors**	***B* (SE)**	**95% CI**	***p***	**Beta**^**1**^	***f***^**2**^** (9`5% C.I.)**^**2**^
PA (difference in hours/week)^a^	Received pedometer (0 = no, 1 = yes)	0.36 (0.59)	−0.81 to 1.54	0.54	0.04	0.00
	Age (in years)	0.04 (0.04)	−0.05 to 0.12	0.41	0.06	0.00
	Gender (0 = male; 1 = female)	−0.18 (0.62)	−1.40 to 1.04	0.77	−0.02	0.00
	Hypertension (0 = no hypertension; 1 = hypertension)	−0.19 (0.62)	−1.41 to 1.03	0.77	−0.02	0.00
	Education level—high (0 = no; 1 = yes)	−1.46 (0.74)	−2.91 to−0.01	** < 0.05**	−0.15	0.02
	Education level—intermediate (0 = no; 1 = yes)	−0.68 (0.75)	−2.16 to 0.79	0.36	−0.07	
	Region (0 = southern Limburg; 1 = northern Limburg/North Brabant)	−2.56 (0.62)	−3.79 to−1.33	** < 0.001**	−0.29	0.09
	***% MI adherent***	*0.03 (0.02)*	*0.00 to 0.06*	** < *0.05***	*0.15*	*0.02*
Intake of fruit (difference in servings/day)^b^	Received pedometer (0 = no, 1 = yes)	0.05 (0.30)	−0.54 to 0.64	0.88	0.01	0.00
	Age (in years)	−0.04 (0.02)	−0.08 to 0.01	0.09	−0.14	0.02
	Gender (0 = male; 1 = female)	−0.15 (0.32)	−0.77 to 0.48	0.65	−0.04	0.00
	Hypertension (0 = no hypertension; 1 = hypertension)	−0.08 (0.32)	−0.70 to 0.54	0.80	−0.02	0.00
	Education level—high (0 = no; 1 = yes)	−0.11 (37)	−0.83 to 0.61	0.77	−0.02	0.00
	Education level—intermediate (0 = no; 1 = yes)	−0.16 (0.38)	−0.92 to 0.59	0.67	−0.04	
	Habit strength (sum of 3 items (*α* = 94), e.g., “Eating fruit is something I do frequently” (1 = completely disagree to 5 = completely agree))	−0.11 (0.05)	−0.20 to−0.01	** < 0.05**	−0.17	0.03
	***% MI adherent***	*0.02 (0.01)*	*0.00 to 0.03*	** < *0.05***	*0.16*	*0.03*
Intake of vegetables (difference in grams/day)^c^	Received pedometer (0 = no, 1 = yes)	11.41 (9.98)	−8.29 to 31.12	0.25	0.08	0.01
	Age (in years)	0.78 (0.73)	−0.66 to 2.22	0.29	0.08	0.01
	Gender (0 = male; 1 = female)	18.31 (10.73)	−2.87 to 39.50	0.09	0.12	0.02
	Hypertension (0 = no hypertension; 1 = hypertension)	21.50 (10.49)	0.79 to 42.21	** < 0.05**	0.15	0.02
	Education level—high (0 = no; 1 = yes)	10.64 (12.18)	−13.41 to 34.68	0.38	0.07	0.02
	Education level—intermediate (0 = no; 1 = yes)	24.68 (12.89)	−0.76 to 50.12	0.06	0.15	
	Habit strength (sum of 3 items (*α* = 91), e.g., “Eating vegetables is something I do frequently” (1 = completely disagree to 5 = completely agree))	5.20 (2.35)	0.56 to 9.83	** < 0.05**	0.18	0.02
	Number of action plans (0–5)	−15.78 (5.10)	−25.74 to−5.72	** < 0.01**	−0.22	0.05
	Stages of change (1 = I have no plans to execute the behavior (not motivated) to 6 = I am executing the behavior for more than 6 months (maintainer))	−7.98 (3.09)	−14.09 to−1.87	** < 0.05**	−0.20	0.04
	Smoking behavior (0 = not smoking; 1 = smoking)	−47.90 (13.07)	−73.69 to−22.11	** < 0.001**	−0.25	0.09
	***Global clinician rating**** SPIRIT (1* = *low to 5* = *high)*	−*34.62 (9.34)*	−*53.05 to*−*16.20*	** < *0.001***	−*0.26*	*0.08*

MI fidelity scores are presented in italics

*PA* physical activity, *CI* confidence interval

*The intervention effects are based on the final regression models

^a^*n* = 190; *R*^2^ = 0.12

^b^*n* = 176, *R*^2^ = 0.08

^c^*n* = 187, *R*^2^ = 0.21

Beta^1^ is the standardized beta

*f*^*2*^ is an effect size index classified as small (≤ 0.02), medium (≤ 0.15), or large (≤ 0.35) (Cohen 1988)

#### Physical Activity

Diverse sociodemographic variables, potential cognitive-behavioral determinants, other lifestyle and health variables, and baseline behavior of other outcomes were added as covariates with PA as the outcome behavior. Direction was positively related (*p *< .05) to change in the level of PA from baseline to 47-week follow-up with a medium effect size. Since the ICC for Direction was not optimal (ICC = .35) [19], the analysis was repeated without Direction. Subsequently, the percentage of MI adherent expressions was significantly (*p *< .05) and positively related to change in the level of PA from baseline to 47-week follow-up, with a small effect size (*f*^2^ = .02; unstandardized *β *= 0.003; standard error = .02, 95% confidence interval = .000 to 0.06; standardized *β *= .15, *p *< .05.

#### Fruit Intake

Improvement in intake of fruit was significantly and positively related to the percentage of MI adherent expressions (*p *< .05), with a medium effect size.

#### Vegetable Consumption

Only the global clinician rating “Spirit” was significantly (*p *< .001), but inversely, related to the change in vegetable consumption from baseline to the first follow-up measurement with a medium effect size. The inverse relation between global counselor rating “Spirit” and vegetable intake was further explored. Whether MI works better in people who are less motivated to change as Stages of Change was checked a predictor of vegetable intake improvement [40]. The interaction between Spirit and Stage of Change was added to the final model with change in vegetable consumption as the outcome. This interaction was not significant (unstandardized *β *= −.50; standard error = 4.85; 95% confidence interval = −10.08 to 9.08; standardized *β *= −.05, *p *= 92). In addition, it was explored whether the inverse relation between “Spirit” and change in vegetable consumption could be explained by the type of diet behavior. The Pearson correlation between baseline fruit and vegetable intake was examined and found unrelated (*r *= .10, *p *= .13). This was similar to the relation between change in fruit and vegetable intake (*r *= .02; *p *= .83).

To examine if the choice of the order of conversation topic in the TMI sessions influenced the relation between MI fidelity summary scores and behavior change, two dummy variables indicating topic choice in conversation one and three (both 0 = PA first; 1 = intake of fruit and vegetables first) were added to the final models described before. The choice of the conversation topic at sessions 1 and 3 was not related to behavior change or behavior at follow-up corrected for baseline behavior (all *p*>.05), and including both dummies as predictors did not change the *B*’s of the fidelity predictors.

## Discussion

This paper aimed to explore the association between telephone MI fidelity using MITI 3.0 indicators and changes in PA and fruit and vegetable consumption. MI adherent expressions seemed significantly and positively related to an increase in PA and fruit intake. MI “spirit” seemed significant, but inversely related to vegetable intake. But, findings need to be interpreted with caution considering multiple testing bias (*p*-value below *p *< .05, but above .01).

Although Direction appeared positively and significantly related to change in the level of PA with a medium effect size, it was omitted from analysis because of the low inter-rater correlation [19]. The reason for this is that it is important to distinguish “competent” from “not competent” delivered intervention elements to be able to determine what is “MI” when studying the association between MI and behavioral change. Other researchers have reported low ICCs for global scores as well and suggested that this may be due to the restricted 5-point scoring range [41]. More research may be needed to investigate the assessment of Direction since it might be relevant to PA change.

Our findings for PA are in line with research showing that MI, in general, is an important predictor for intervention effects [42]. Also, other research revealed that MI adherent counseling behaviors were associated with increases in PA behavior [43, 44].

Comparing our nutrition findings to those of other research and reviews is complicated by the fact that many studies and reviews combine fruit and vegetable intake into one outcome, e.g., [45, 46]. Although in our study MI fidelity was positively related to fruit increase, the opposite was true for vegetable intake. The unexpected negative influence of MI fidelity on vegetable consumption was further examined, especially since our TMI intervention was shown to outweigh the control condition for this behavior [30]. Stage misclassification may explain this finding and has been shown in other studies to be more substantial for vegetable intake than for fruit intake [47]. Stage of change, as MI is thought to be especially effective in unmotivated participants [48], did not explain this finding. As vegetables are more often eaten in mixed dishes than fruit and therefore more difficult to conceptualize in terms of servings [9], it may have happened that when counselors stimulated autonomy of and exploration by participants regarding their vegetable intake, the phenomenon *the more one thinks about it*, *the more unclear it becomes* occurred. The complexity of the “vegetable” behavior might have hindered the client’s behavior change process. Our study is not the only one that has revealed contra-intuitive findings regarding MI fidelity and “vegetable” intake. A recent review reported smaller MI effects on FVC in reports where researchers reported greater consistency with the MI style [49]. Unfortunately, fruit and vegetable intake were not reported separately.

In our telephone sessions, fruit and vegetable intake were discussed together, starting with fruit intake. Both behaviors improved in the intervention condition compared to control. A review showed that effect sizes for combined FVC intake are bigger when sessions take 30 to 40 min [49]. Ours were 20 min on average. Maybe that during the short conversation vegetable intake was lost sight of to some extent. Counselors who scored high on MI spirit (evocation, autonomy support, collaboration) may have been less successful in achieving vegetable intake change because they never reached the point to discuss “action” compared to counselors who more quickly moved to this stage of the change process. That fruit classification is less debated than vegetable classification [50] may also imply that vegetable intake is a relatively more complex behavior. Changing such behavior may have been helped by expert advice to increase the behavioral capability. However, to check these ideas, the content of the recordings will need to be reassessed.

Vegetable intake and fruit intake appeared unrelated. The latter might again point out that these are indeed distinct behaviors that should probably not automatically be combined into one combined fruit vegetable consumption (FVC) outcome. As stated, discussing changing two distinct behaviors at the same time during a telephone conversation might be too much to handle. Results of whether it is better to discuss fruit and vegetables sequentially or simultaneously without overloading participants remain inconclusive [51]. More research into the separate relation between MI fidelity and intake of vegetables is needed, including the search for a hidden confounder which may have affected vegetable consumption, but not fruit consumption or PA in the present study.

The strength of this study is that almost all conversations were recorded, the large number of MITI-assessed motivational interviews and the computer-generated randomly selected study sample, representing a substantial group of counselees (low selection bias). The randomization and random selection as conducted in this study also enable the generalization of the study findings, especially to different samples and cultural context. Sessions were selected from different time points with diverse clients to have a representative view of counselors MI skills and patients at different stages of change [21]. Besides, coders could not foresee which interviews they had to code (low selection bias) and were blind to the lifestyle change outcomes (low performance and detection bias). Dropout was low and unlikely to be related to the true outcome (low attrition bias). Fruit intake, vegetable consumption, and physical activity have consistently been found to be positively correlated, e.g., [52]. This study accounted for such transfer effects by including change in other lifestyle behaviors in the evaluation of potential predictors of change for a particular behavior. Most studies that explored the association of MI with behavior change focused on addictive behaviors (alcohol consumption, drug use, or smoking), e.g., [53, 54]. In this study, the relation between MI and behavior change was examined outside the addiction field. Finally, by including covariates in our analyses, power and precision of predictor testing and estimation are improved due to reduced residual outcome variance [39].

Yet, some limitations need to be addressed. MI fidelity scores of our counselors were generally at the beginning proficiency level. It is still unclear what level of expertise is required for MI to work [55]. Nevertheless, a link between some MI fidelity aspects and lifestyle behavior change was found. Whether higher fidelity will lead to more behavior change is still to be established. Findings suggested that participants in the randomly selected interviews were more likely to live together with a partner than other participants. This may have biased the relation between MI fidelity codes and behavior change. However, marital status was not a significant covariate in the analyses relating MI fidelity to lifestyle change, and because of multiple testing in the selection check (the *p*-value for marital status was .04), there seems to be no evidence for selection bias in our results. MI fidelity was assessed to ensure that our intervention is true to the character of MI to avoid error when drawing conclusions from our results. The MITI 3.0 is one of the tools available to assess MI fidelity [56, 57]. With this tool, the impact of MI features on client conversation behavior, for instance, was not assessed (e.g., change talk or sustain talk). Yet, some indicators of “client behaviors” such as self-reported self-efficacy were included in the analysis as covariates. Nevertheless, associations with client behavior may differ when using other fidelity measures [56]. Furthermore, the focus was on the relation between MI fidelity and change in three lifestyle behaviors. The MITI 3.0 scores were averaged across different sessions and therapists. Meantime, other studies have shown that MI fidelity varies between sessions and therapists [58]. Future studies should therefore also perform more refined analyses to determine variability between session and therapists regarding MI fidelity and the relation with behavior change [21]. In this study, the MI fidelity was rated concerning selected sessions. Other than that the coders involved had no relation with the MI counselors, bias in individual raters was not assessed (as halo, horn, leniency, or central tendency errors) [59]. Thus, due to rater bias as well as random error, caution is advised in the interpretation of the MITI scores. Importantly, competence in MI was tied to self-reported client improvement. The use of self-report methods to assess PA, fruit intake, or vegetable intake is a potential limitation. It may be beneficial to use both self-report and objective measures in future studies. Furthermore, this study occurred in specific regions of The Netherlands and used telephone-based guided MI, all of which may limit the external validity of our results. Although our study revealed significant changes in lifestyle behavior, only a few aspects of MI could be cautiously related to these increases in the final model. This may be due to the suboptimal levels of MI treatment integrity for some aspects as shown by our assessment study [19]. Hence, it needs to be noted that several other studies have shown no relation between a higher competence level of the MI interventionist and better study outcomes, e.g., [53, 54, 60]

## Conclusion

This study moves the scientific field forward because it showed that particular MI counselor behaviors, as assessed with the MITI 3.0, were predictive of behavioral lifestyle outcomes. Such findings contribute to the unraveling of the potential mechanisms of change regarding these behaviors [47], and, as such, inform practice, processes, and training of MI. As our counselors had beginner proficiency, there is potential for improvement and a need for more careful MI training and monitoring of fidelity. MI adherent responses improved PA and fruit intake. Higher “spirit” scores hindered vegetable intake. Further studies exploring the relationship between MI “spirit” and vegetable intake separate from fruit consumption are needed since others found a positive relationship with “spirit” when addressing fruit and vegetable intake as a combined outcome [61].
